# Microcutting Redox Profile and Anatomy in *Eucalyptus* spp. With Distinct Adventitious Rooting Competence

**DOI:** 10.3389/fpls.2020.620832

**Published:** 2021-01-20

**Authors:** Johnatan Vilasboa, Cibele Tesser da Costa, Leonardo Girardi Ransan, Jorge Ernesto de Araújo Mariath, Arthur Germano Fett-Neto

**Affiliations:** ^1^ Plant Physiology Laboratory, Center for Biotechnology and Institute of Biosciences (Department of Botany), Federal University of Rio Grande do Sul, Porto Alegre, Brazil; ^2^ Plant Anatomy Laboratory, Institute of Biosciences (Department of Botany), Federal University of Rio Grande do Sul, Porto Alegre, Brazil

**Keywords:** rooting, Eucalyptus, reactive oxygen species, antioxidant defense, wounding, xylem, flavonoid

## Abstract

Adventitious root (AR) development takes place in an intricate cellular environment. Reactive oxygen species (ROS) and antioxidant defenses, triggered by wounding in cuttings, can modulate this process. A comparative assessment of biochemical and anatomical parameters at critical rooting stages in hard- (*Eucalyptus globulus* Labill.) and easy- (*Eucalyptus grandis* W.Hill ex Maiden) to-root species was carried out. Microcuttings from seedlings were inoculated in auxin-free AR induction medium and, after 96 h, transferred to AR formation medium for a period of 24 h. Samples were collected upon excision (Texc) and at the 5th day post excision (Tform). Delayed xylem development, with less lignification, was recorded in *E. globulus*, when compared to *E. grandis*, suggesting lower activity of the cambium layer, an important site for AR development. Superoxide was more densely present around the vascular cylinder at both sampled times, and in greater quantity in *E. globulus* than *E. grandis*, declining with time in the former. Hydrogen peroxide was localized primarily along cell walls, more intensely in the primary xylem and phloem, and increased significantly at Tform in *E. globulus*. Ascorbate peroxidase (APX), superoxide dismutase (SOD), and catalase (CAT) activities were generally higher in *E. grandis* and varied as a function of time in *E. globulus*. Soluble guaiacol peroxidase (GPRX) activity increased from Texc to Tform in both species, whereas cell wall-bound GPRX activity increased with time in *E. grandis*, surpassing *E. globulus*. Flavonoid content increased with time in *E. grandis* and was higher than *E. globulus* at Tform. Principal component analysis showed that species- and time-derived differences contributed to almost 80% of the variance. Overall, data indicate that *E. grandis* shows higher cambium activity and tighter modulation of redox conditions than *E. globulus*. These features may influence ROS-based signaling and phytohormone homeostasis of cuttings, thereby impacting on AR development. Besides being players in the realm of AR developmental differences, the specific features herein identified could become potential tools for early clone selection and AR modulation aiming at improved clonal propagation of this forest crop.

## Introduction

Adventitious roots (ARs) are formed post-embryonically from stems, leaves, hypocotyls, and other organs or tissues where previously there were no roots (Bellini et al., 2014; Steffens and Rasmussen, 2016). AR development is a complex process that involves several phytohormones, taking part in different steps of AR formation (Da Costa et al., 2013). Auxins, however, have proven to be central players acting synergistically or antagonistically with other phytohormones to stimulate AR initiation and development (Pacurar et al., 2014). Two main steps are recognized in AR formation. The first is known as the induction phase, in which high auxin levels are required and biochemical changes take place. This stage is followed by the formation phase, when cell division and differentiation occur, leading to AR primordium development and, finally, AR emergence (Da Costa et al., 2013). AR formation occurs naturally in some species, mainly in monocots, in which the primary root system is important for seedling development, but is shortly replaced by ARs, forming a fibrous root system (Bellini et al., 2014). Still, various conditions can induce it, such as flooding, etiolation, burial, soil chemicals, nutrient deficiency, or wounding (Steffens and Rasmussen, 2016).

The physical damage caused by wounding triggers several biochemical and physiological signals that lead to a systemic response. Reactive oxygen species (ROS), initially perceived only as toxic, have been shown to act as important signal transduction molecules in wound response (Mittler et al., 2011). The balance between toxic, non-toxic, or signaling depends on the action of ROS-scavenging mechanisms. These modulators can detoxify excessive ROS derived from cellular metabolism or ROS-producing enzymes (Mittler et al., 2004). Enzymatic antioxidants such as superoxide dismutase (SOD), catalase (CAT), ascorbate peroxidase (APX), and guaiacol peroxidase (GPRX) play important roles in ROS-scavenging capacity. SOD helps maintain the redox balance by the dismutation of superoxide radical (O_2_^•−^) into hydrogen peroxide (H_2_O_2_) and molecular oxygen (O_2_; Kapoor et al., 2019). APX and CAT are fundamental to detoxify and maintain adequate cellular levels of H_2_O_2_ for normal plant growth and development (Anjum et al., 2016). APX uses ascorbic acid as an electron donor to detoxify H_2_O_2_, whereas CAT can directly degrade it. GPRX uses several substrates as reducers to eliminate H_2_O_2_ but can also produce ROS (Gechev et al., 2006). Flavonoids are a group of plant phenolics with non-enzymatic antioxidant properties, which can inactivate ROS generated during abiotic stress (Vicente and Boscaiu, 2018).

Participation of ROS in adventitious rooting is still poorly understood. Some lines of evidence indicate that H_2_O_2_ might act as a signaling molecule, inducing AR formation (Li et al., 2009; Huang et al., 2020). In this process, auxin is involved and thought to modulate AR growth through ROS accumulation, which is counterbalanced by a mutual regulation between ROS, nitric oxide (NO), and auxin to maintain cell redox homeostasis and signaling (Correa-Aragunde et al., 2016).

Wound-induced roots are especially important for plant propagation of economically relevant species in forest and horticulture industries (Steffens and Rasmussen, 2016; Vilasboa et al., 2018). This is the case of eucalyptus propagation, which is mainly vegetative and widely employed in the commercial forest industry for uniform cutting production from select superior genotypes. Eucalypt trees serve as one of the major sources of wood for timber, pulp, cellulose, and paper industries worldwide (Vilasboa et al., 2018). Eucalypt wood has also emerged as feedstock for chemicals, fuels, and other materials through biorefinery technologies (Penín et al., 2020). Out of ca. 900 known *Eucalyptus* species, *Eucalyptus globulus* stands out for its high pulp yield, wood density, and fiber quality (Carrillo et al., 2018). Low lignin levels and high syringyl/guaiacyl (S/G) residue ratio contribute to the high pulp yield (Rencoret et al., 2007). However, vegetative propagation of this species and some of its hybrids is often limited due to its relatively low ability to produce ARs (De Almeida et al., 2015).

Although it is known that ROS can play important roles in AR development, this oxidative component has not been examined in detail in *Eucalyptus* cuttings. In this work, an *in vitro* system was used to perform a morphological and biochemical comparison of the easy-to-root *Eucalyptus grandis* and hard-to-root *E. globulus* species in an attempt to better understand the dynamics of wound induced ROS and AR formation. The hypotheses tested were: (i) Microcutting anatomy features are related to root regeneration capacity; (ii) particular changes in ROS-related parameters of cuttings occur between the time of severance and early AR development; and (iii) distinct profiles of ROS-related parameters are apparent in eucalypt species of contrasting root regeneration capacity. The range of assessed parameters revealed specific patterns associated with each species and stage of AR development and may provide tools to characterize and possibly modulate rooting recalcitrance.

## Materials and Methods

### Plant Material

*Eucalyptus globulus* Labill. and *E. grandis* W.Hill ex Maiden were used in the experiments. Seed surface sterilization and germination procedures were carried out according to De Almeida et al. (2015), with minor modifications. Surface-sterilized seeds were placed in groups of 15 distributed in approximately 30 to 40 glass jars of 300 ml containing 60 ml of half-strength MS salts (Murashige and Skoog, 1962) at pH 5.8 and 0.7% agar. Before sowing, jars were capped with a double layer of aluminum foil, autoclaved at 121°C for 20 min. After sowing, jars were sealed with plastic film at the limit of foil and glass surface. Seedlings were kept under 16 h photoperiod of 100 μmol m^−2^ s^−1^ photosynthetically active radiation (provided by white LED lights) and temperature of 23 ± 2°C.

### Adventitious Rooting

For *in vitro* adventitious rooting, 3-cm apical microcuttings with only two fully expanded leaf pairs were obtained by excision with a scalpel blade (Supplementary Figure 1). Remaining leaves were excised. Since the two species grow at different rates, *E. globulus* was excised with 14 weeks and *E. grandis* with 16 weeks after sowing, unless otherwise stated. The rooting culture system comprised two sequential steps, essentially as previously described (Fett-Neto et al., 2001; De Almeida et al., 2015) with modifications. First, cuttings were kept in induction medium containing 0.3x MS salts, 40 mg l^−1^ thiamine-HCl, 100 mg l^−1^ myo-inositol, and 0.6% agar for 96 h. Then, cuttings were transferred to formation medium, identical in composition but with the addition of 0.1% activated charcoal. Media were devoid of sucrose and auxin, and pH was adjusted to 5.8 before autoclaving at 121°C for 20 min. Adventitious rooting experiments were carried out in 20 ml glass vials containing 6 ml of medium and one cutting. Vials were sealed and kept under controlled conditions as mentioned in Plant Material section.

### Sampling

Microcuttings were harvested at the moment of excision (0 h induction stage, henceforth Texc) and 24 h after the transfer to formation medium (24 h into the formation stage, henceforth Tform). The latter harvest point was chosen as it generally coincides with early AR primordia development (De Almeida et al., 2015). For biochemical analyses, samples were weighed, immediately frozen in liquid nitrogen and stored at −80°C. Each biological replicate was composed of five whole microcuttings and five replicates were used. For morphological analyses, plants were kept in formation medium for 20 days. Three independent experiments were performed (*n* = 20) to quantify rooting percentage, length of the longest root, and root number. For anatomical analysis and lignin staining, microcutting stem bases were fixed under vacuum in McDowell and Trump’s fixative solution of 4% formaldehyde and 1% glutaraldehyde in sodium phosphate buffer, pH 7.2 (McDowell and Trump, 1976), and kept at 4°C. For ROS staining, microcutting stem bases were immediately used for histological sectioning in a hand microtome (Ranvier type) with the help of a soft wedge to hold samples in place. Twelve replicates were used for anatomical evaluations with lignin staining and three for ROS localization.

### Lignin Staining

Cross sections of 10–15 μm were obtained from fixed material using a handheld microtome. Histological sections were kept in distilled water to avoid dehydration and then transferred to spot plates for staining with phloroglucinol and HCl, according to Johansen (1940), with minor modifications. A few drops of 1% phloroglucinol in 95% ethanol [v/v] were applied to the sections, followed by a few drops of 30% HCl. After 5 min, slides were prepared in water and images were immediately captured. The histological sections were observed using a Leica DMR microscope, coupled with digital image capture system DFC500 (Leica), using 20x or 40x long working distance objective lenses.

### ROS Localization

Protocols for ROS visualization were adapted from Romero-Puertas et al. (2004). Histological cross sections were obtained as mentioned in Lignin staining section from fresh microcutting bases. Immediately, sections were transferred from distilled water to 1 mM ascorbic acid in spot plates for 10 min. Then, for superoxide staining, sections were immersed for 1 h in a solution of 1.41 mg ml^−1^ nitro blue tetrazolium (NBT), after which dark blue spots indicated NBT reduction. For H_2_O_2_ staining, separate sections were immersed in 1 mg ml^−1^ 3,3'-diaminobenzidine (DAB) in 50 mM phosphate buffer (pH 7.0) for 30 min under LED lighting. Oxidized DAB produces a distinct brownish precipitate. For chlorophyll removal, sections were then transferred to 95% ethanol [v/v] for 15 min. Slides were prepared in water and images were obtained as described in Lignin staining section.

### Image Processing and Area Measurements

All area measurements of images obtained through photomicrography were done using ImageJ2 v1.53c (Rueden et al., 2017) and distribution Fiji (Schindelin et al., 2012). Xylem area percentage (*n* = 12) was defined as the area ratio of xylem tissue and the whole section. NBT- and DAB-stained area percentages were estimated using the Color Threshold feature of ImageJ2.

### Enzyme Activity Assays

Total protein extract was obtained from approximately 100 mg of frozen plant tissue. Samples (*n* = 5) were ground in liquid nitrogen and solubilized in 1.5 ml extraction buffer [50 mM HEPES – pH 7.4, 1% polyvinylpyrrolidone, 1 mM EDTA, and 0.1% Protease Inhibitor Cocktail (Sigma, United States)]. After centrifugation at 12,800 × *g* at 4°C for 15 min, the supernatant was recovered and immediately used for enzymatic activity assays. Protein content was determined according to Bradford (1976). Spectrophotometric readings were obtained with Spectramax M2 Microplate Reader (Molecular Devices, United States).

Ascorbate peroxidase activity was assayed according to Nakano and Asada (1981). Reaction mixture contained 2 ml of buffer (50 mM phosphate buffer – pH 7.0, 0.5 mM ascorbic acid, and 1 mM H_2_O_2_) and 50 μl of the total protein extract (~56 μg protein). Changes in absorbance due to ascorbic acid oxidation (using *ε* = 2,800 M^−1^ cm^−1^) were monitored at 290 nm.

Two fractions of guaiacol peroxidase were assayed, following different extraction protocols. Cell wall-bound GPRX activity was measured using an extract enriched for cell wall-bound protein, as described in Dos Santos et al. (2004). Briefly, pellets obtained after supernatant recovery were profusely washed until no soluble GPRX activity could be detected. Pellets were then incubated in 1 ml 1 M NaCl at 4°C for 1 h. The supernatant recovered after 15 min centrifugation at 10,000 × *g* was used for activity determination as described by Cakmak and Marschner (1992). To 1.5 ml of reaction buffer (50 mM phosphate buffer – pH 7.0, 0.05% guaiacol, and 10 mM H_2_O_2_), 20 μl of total extract (~22 μg protein) or cell wall-bound enriched extract were added. Tetraguaiacol formation (using *ε* = 26,600 M^−1^ cm^−1^; Chance and Maehly, 1955) was monitored at 470 nm.

Catalase activity was determined (Cakmak and Marschner, 1992) in a reaction mixture comprising 1.5 ml of the reaction buffer (25 mM phosphate buffer – pH 7.0, and 10 mM H_2_O_2_) and 20 μl of total protein extract (~22 μg protein). H_2_O_2_ decomposition (using ε = 39.4 M^−1^ cm^−1^; Nelson and Kiesow, 1972) was followed at 240 nm.

Superoxide dismutase activity was measured as per Beyer and Fridovich (1987). To 1 ml of the reaction buffer (50 mM phosphate buffer – pH 7.8, 57 μM NBT, 9.9 mM L-methionine, and 0.025% Triton X-100), 10 μl of 44 mg l^−1^ riboflavin and 20 μl of total protein extract (~11 μg protein) were added. The reaction took place under white light for 15 min at room temperature. Final absorbance value was read at 560 nm. One SOD activity unit (U) was defined as the amount of active enzyme needed to inhibit NBT reduction by 50%.

### Flavonoid Content

Flavonoid content was estimated according to Zhishen et al. (1999) with minor modifications. Approximately 40 mg of frozen plant tissue was ground in liquid nitrogen, extracted in 300 μl of 95% ethanol [v/v], and placed in an ultrasonic bath for 30 min at 4°C in the dark. The next steps were performed under indirect light. Extracts were centrifuged at 15,000 × *g* for 10 min at 4°C and 100 μl of the supernatant were mixed with 400 μl of distilled water and 30 μl of 5% NaNO_2_ and kept at room temperature for 5 min. Then, 30 μl of 1% AlCl_3_ were added and vigorously mixed. After 6 min at room temperature, 200 μl of 1 M NaOH and 240 μl of distilled water were added and mixed well. The absorbance was measured at 510 nm and a standard curve was established using quercetin (Sigma, United States).

### Statistical Analysis

Means were compared by Student’s paired *t*-test using GraphPad Prism v8.0 for Windows (GraphPad Software, United States), unless stated otherwise. Data were expressed as mean ± SEM and statistical significance was set at *p* ≤ 0.05.

For principal component analysis (PCA), log-transformed data from three biological replicates for each species (sp.) × time permutation in respect to nine assessed variables were used. Factors were retained according to the Kaiser criterion, i.e., eigenvalue > 1. PCA was performed in R 4.0.3 (R Core Team, 2020), using packages “factoextra” (Kassambara and Mundt, 2020), “FactoMineR” (Lê et al., 2008), “corrplot” (Wei and Simko, 2017), and “pca3d” (Weiner, 2020).

## Results

### *E. grandis* Shows Higher Rooting Capacity and Xylem Development When Compared to *E. globulus*

**Figure 1:**
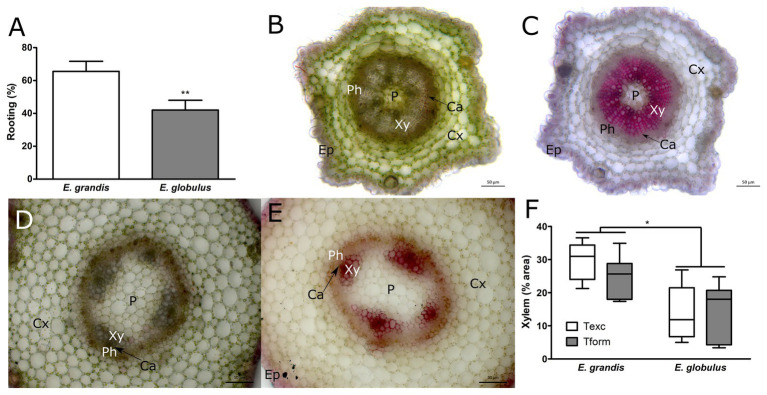
Morphological and developmental patterns of *Eucalyptus* spp. microcuttings. **(A)** Rooting capacity (%) of *Eucalyptus grandis* and *Eucalyptus globulus* microcuttings (*n* = 60, ^**^ *p* < 0.01, paired *t*-test). Unstained stem transversal sections **(B,D)** and lignin stained **(C,E)** of *E. grandis* **(B,C)** and *E. globulus* **(D,E)**. Images of stem transversal sections were taken at the time of excision (P, pith; Xy, xylem; Ca, cambium; Ph, phloem; Cx, cortex; and Ep, epidermis). **(F)** Xylem tissue area percentages found in *E. grandis* and *E. globulus* transversal stem sections, at the time of excision (Texc) and at the formation stage of adventitious root (AR) development (Tform; *n* = 12, ^*^ *p* < 0.05, paired *t*-test). Scale bar = 50 μm.

As previously reported, *E. grandis* showed higher rooting capacity when compared to *E. globulus* (Figure 1A), yielding similar number of roots per microcutting and root system length (Supplementary Figure 2). Freshly cut sections showed an overt difference in xylem size. *E. grandis* displayed a more pronounced degree of xylem development (Figure 1B), whereas *E. globulus* appeared somewhat delayed in its development, often showing various degrees of stellate tissue organization (Figure 1D). In fact, after image processing and xylem area quantification, this finding proved to be statistically significant (Figure 1F). As shown in Figure 1D, *E. globulus* xylem was often restricted to four poles, barely forming a vascular ring, whereas a well-defined ring was the predominant feature in *E. grandis* microcuttings. Xylem area did not significantly differ between sampling times for either species.

Xylem is highly lignified, and *E. globulus* trees are in the lower end of *Eucalyptus* spp. lignin content (Nawawi et al., 2017). To investigate if such difference in development could be correlated with lignin content, microcuttings were stained with phloroglucinol (Figures 1C,E). Additionally, whole-cutting lignin content was measured using acetyl bromide method. Phloroglucinol-stained area percentages largely overlapped with xylem area percentages. However, whole-cutting lignin content did not change between species or sampling times (data not shown). At Tform, some AR primordia were forming inside microcutting stems (Supplementary Figure 3).

**Figure 2:**
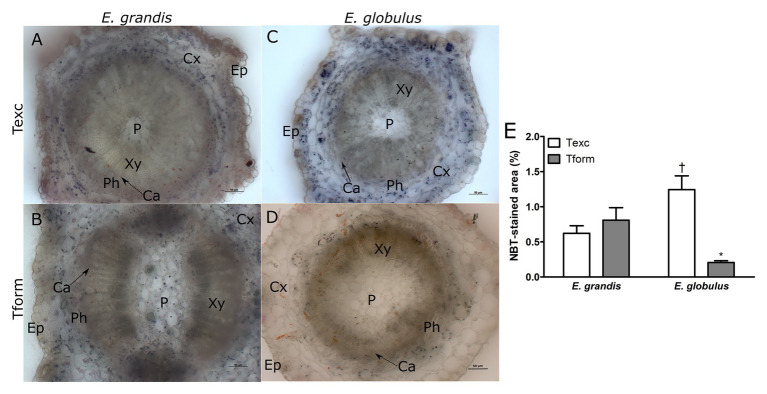
Superoxide localization in *Eucalyptus* spp. microcutting stem bases. *E. grandis* **(A,B**) and *E. globulus* **(C,D)** nitro blue tetrazolium (NBT)-stained microcutting transversal stem sections at the time of excision – Texc **(A,C)** and at the formation stage of AR development – Tform **(B,D**; P, pith; Xy, xylem; Ca, cambium; Ph, phloem; Cx, cortex; and Ep, epidermis). **(E)** NBT-stained area percentage in *E. grandis* and *E. globulus* transversal stem sections at Texc and Tform (^*^ *p* < 0.05, paired *t*-test between time points; ^†^ *p* < 0.05, paired *t*-test between spp.). Scale bar = 50 μm.

**Figure 3:**
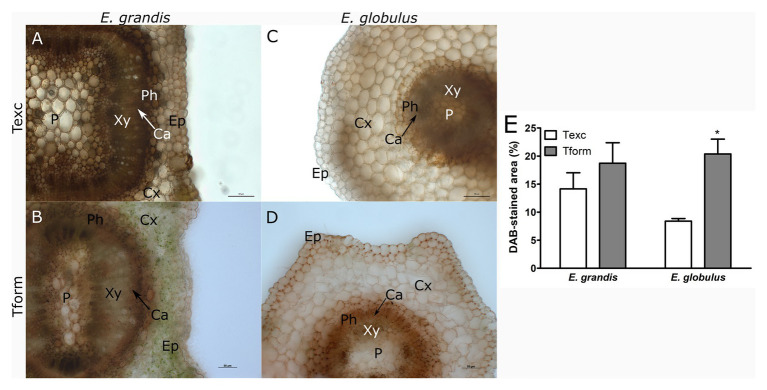
Hydrogen peroxide localization in *Eucalyptus* spp. microcutting stem bases. *E. grandis* **(A,B)** and *E. globulus* **(C,D)** microcutting 3,3'-diaminobenzidine (DAB)-stained transversal stem sections at the time of excision – Texc **(A,C)** and at the formation stage of AR development – Tform **(B,D**; P, pith; Xy, xylem; Ca, cambium; Ph, phloem; Cx, cortex; and Ep, epidermis). **(E)** DAB-stained area percentage in *E. grandis* and *E. globulus* transversal stem sections at Texc and Tform (^*^ *p* < 0.05, paired *t*-test between time points). Scale bar = 50 μm.

Both species displayed a range of growth phenotypes, especially since microcuttings were seed-derived, adding variability to the samples. It is worth noting that there was remarkable variation in xylem development, especially in *E. globulus*. As per Figure 1F, there was some overlap between species, as shown in Figures 2, 3. *E. globulus* has a higher mean growth rate than *E. grandis*. Accordingly, previous investigations that used this culture system included a 2-week gap between species (as described in Adventitious rooting section) to obtain more uniform microcuttings. To ensure that differences in xylem area percentage were not a matter of growth, 16-week *E. globulus* microcuttings were also assessed. Mean xylem area percentage was equal to 14-week *E. globulus* and inferior to 16-week *E. grandis* (Supplementary Figure 4). This supported the hypothesis that such difference is likely an intrinsic feature of these species.

### ROS-Associated Distribution Patterns Vary in *E. globulus* Along AR Development

Nitro blue tetrazolium staining was used for histochemical detection of superoxide on stem base sections of *E. grandis* and *E. globulus*. At Texc, larger portions of *E. globulus* microcutting sections were stained with NBT than in *E. grandis* (Figures 2A,C,E). Conversely, at Tform, very few stained spots could be detected on *E. globulus* sections (Figure 2D), while no significant difference between sampling times was seen in *E. grandis* (Figures 2A,B,E). In NBT-stained sections, superoxide anion was most frequently found across the cortical region and, in some cases, more adjacent to the vascular tissues (Figures 2A–D).

To study H_2_O_2_ distribution, *E. grandis* (Figures 3A,B) and *E. globulus* (Figures 3C,D) sections were stained with DAB. DAB-stained area did not vary between species at Texc (Figure 3E). *E. globulus* experienced an increase in H_2_O_2_ distribution from Texc to Tform, especially around the outermost domain of phloem tissue (Figures 3C–E). Unlike superoxide, staining could be observed as a continuum. Though some cells displayed a uniformly stained cytoplasm, DAB precipitate could more intensely be detected along cell walls.

While *E. grandis* maintained a similar profile at both time points, ROS distribution in *E. globulus* changed significantly. Superoxide-associated area decreased as H_2_O_2_-associated area increased.

### Specific Changes in Redox-Associated Parameters Hint at Divergent Redox-Associated Patterns

**Figure 4:**
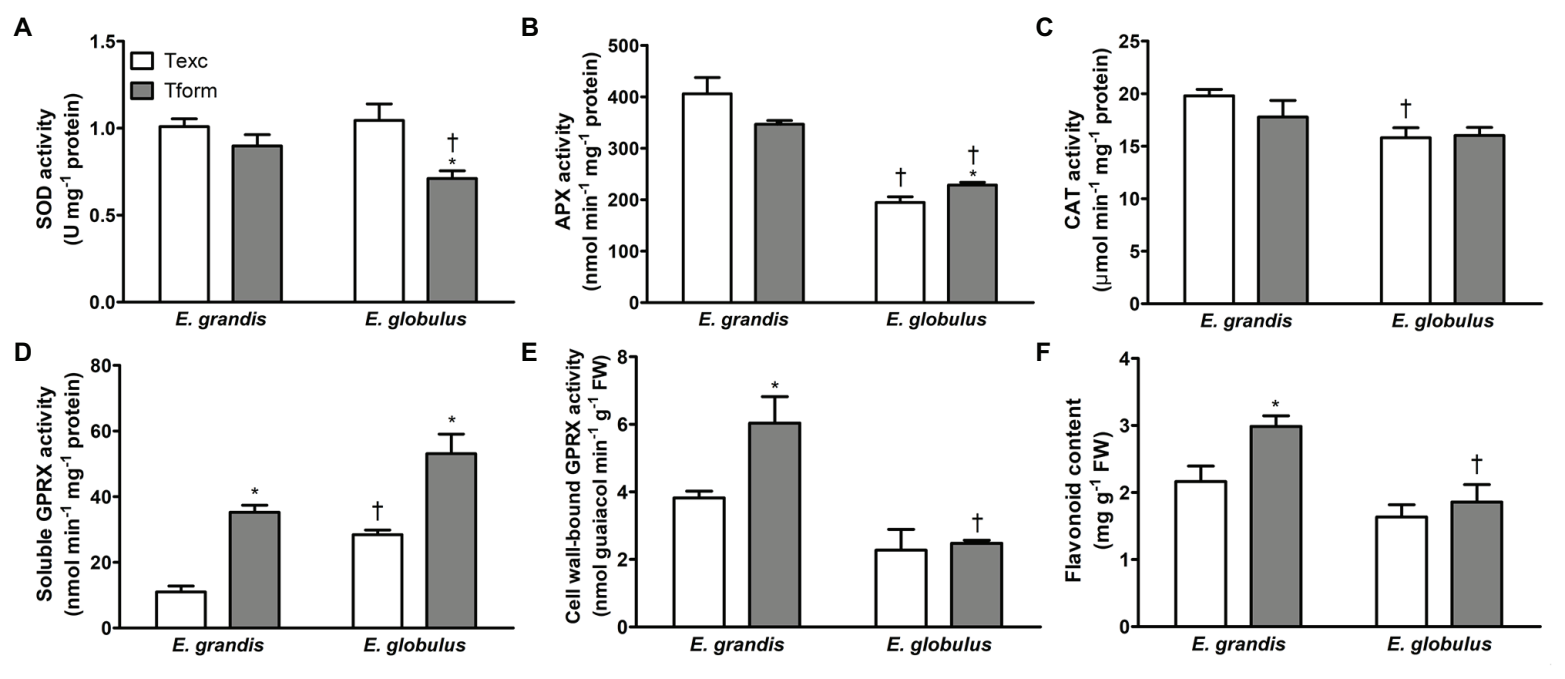
Redox-associated biochemical parameters during adventitious rooting in *Eucalyptus* spp. microcuttings. Superoxide dismutase (SOD; **A)**, ascorbate peroxidase (APX; **B)**, catalase (CAT; **C)**, soluble **(D)** and cell wall-bound guaiacol peroxidase (GPRX; **E)** activity and flavonoid content **(F)** in *E. grandis* and *E. globulus* microcuttings at the time of excision (Texc) and at the formation stage (Tform) of AR development (*n* = 5, ^*^ *p* < 0.05, paired *t*-test between time points; ^†^ *p* < 0.05, paired *t*-test between spp.).

Tissue development and ROS localization are complex phenomena that receive inputs from several genetic and environmental factors. To better characterize the redox status of microcuttings in each AR stage monitored, the activity of some redox-related enzymes was assayed (Figure 4).

Superoxide dismutase activity did not differ between species at Texc, but it decreased with time in *E. globulus* and at Tform was lower than in *E. grandis* (Figure 4A). *E. globulus* APX activity increased from Texc to Tform, but at both sampling times was inferior to that of *E. grandis* (Figure 4B). At Texc, CAT activity was lower in *E. globulus* but did not vary with time in either species (Figure 4C).

Soluble GPRX activity significantly increased between sampling times in both species. At Tform, it was higher in *E. globulus* (Figure 4D). Cell wall-bound GPRX activity, conversely, only increased with time in *E. grandis*, reaching levels higher than those of *E. globulus* at Tform (Figure 4E). Besides the enzymatic component of microcutting antioxidant defense, flavonoid content was assessed as a potential broad-acting ROS quencher (Mierziak et al., 2014). It increased between Texc and Tform in *E. grandis*, and at the latter time point was higher than in *E. globulus* (Figure 4F). The data clearly indicated varied redox profile between the species.

### Principal Component Analysis Identifies Parameters Associated With Species and Rooting Stage

To better understand where both species (at each time point) stand amid the various quantitative parameters, and if the overall picture may be associated with the divergent rooting capacity, a PCA was performed. Nine variables, i.e., xylem percentage area (Xylem%), DAB-stained percentage area (DAB-area), and NBT-stained percentage area (NBT-area) in histological sections, SOD, APX, CAT, soluble GPRX (SolGPRX), cell wall-bound GPRX (CWGPRX) activities, and flavonoid content (flav) in microcuttings were used in the analysis.

**Figure 5:**
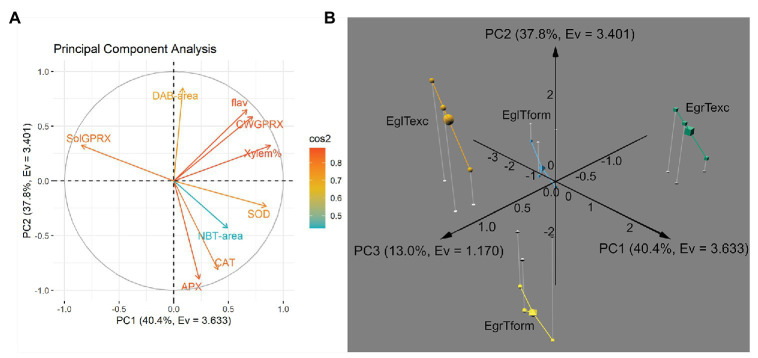
Principal component analysis of histological, histochemical and biochemical parameters assessed during AR development in *E. grandis* and *E. globulus* microcuttings. **(A)** Loadings plot of variables along principal components PC1 and PC2, colored according to cos^2^ values (flav, flavonoid content; CWGPRX, cell wall-bound guaiacol peroxidase activity; Xylem%, xylem area percentage; SOD, superoxide dismutase activity; NBT-area, nitro blue tetrazolium-stained area percentage; CAT, catalase activity; APX, ascorbate peroxidase activity; SolGPRX, soluble guaiacol peroxidase activity; and DAB-area, 3,3'-diaminobenzidine-stained area percentage). **(B)** Individual position on a 3D plot along axes PC1, PC2 and PC3 of *E. grandis* and *E. globulus* three biological replicates (and each corresponded centroid, represented as a larger solid) at the time of excision -Texc (green cubes and orange spheres, respectively) and at the formation stage of AR development -Tform (yellow octahedrons and blue pyramids, respectively). Principal components are expressed as PC [%variance, eigenvalue (Ev)]. EgrTexc, *E. grandis* at Texc; EgrTform, *E. grandis* at Tform; EglTexc, *E. globulus* at Texc; and EglTform, *E. globulus* at Tform.

Figure 5A shows the loadings plot, with color-coded variables according to cos^2^ values (complete version available in Supplementary Figure 5). From cos^2^ values and their ratio to the sum of cos^2^ values for any given principal component (PC), the contribution (%) of each variable to each PC can be estimated (Supplementary Figure 6). Besides, how well represented each variable is in either PC, how strongly these datasets are correlated can provide relevant information (Supplementary Figure 7).

“Xylem%,” “SOD,” SolGPRX,” and “CWGPRX” were the main contributors to PC1. Variance explained by PC2 was mostly made up of “APX,” “DAB-area,” and “CAT.” Interestingly, the variable “flav” split almost evenly between PC1 and PC2. “NBT-area” was not well represented in PC1 and PC2; in fact, it primarily constituted PC3, followed by “SOD” and “SolGPRX.” Together, the first three principal components accounted for over 91% of data variance.

A 3D plot containing data from both species at each time point plotted along the three retained PCs is shown in Figure 5B. Observations derived from three biological replicates of each one of the four sp. × time permutations clustered by group in different octants altogether. Notably, data from both species at Texc clustered on the positive side of the PC2 axis, though in diagonally opposite quadrants on the PC1 × PC3 plane. The same occurred for data from both species at Tform, but on the negative side of the PC2 axis. Thus, this dimension appeared to be more associated with the temporal aspect of AR. Each species clustered to either side of PC1. Hence, this axis carries information about what separates *E. globulus* and *E grandis* regardless of sampling time. PC3 exhibited what differentiates the states of a species at Texc and at Tform.

Overall, the main variables that explain the distance between *E. grandis* and *E. globulus* – irrespective of time – were xylem area percentage, SOD, as well as soluble and cell wall-bound GPRX activity (Supplementary Figure 5). While “SOD,” “Xylem%,” “CWGPRX,” and “flav” had strong positive correlation with PC1 (and, therefore, with *E. grandis* phenotype), “SolGPRX” showed strong negative correlation with PC1 (thus associated with *E. globulus*; Supplementary Figure 7).

Ascorbate peroxidase and CAT activities and DAB-stained area were the main contributors to divergent profiles at Texc and Tform – irrespective of species. PC2 had strong positive correlation with “DAB-area,” “CWGPRX,” and “flav,” and strong negative correlation with “APX” and “CAT.” The last two covariates in this dataset and were strongly associated with Tform. Curiously, flavonoid content contributed almost evenly to the differentiation of species and time (Supplementary Figure 6).

Finally, NBT-stained area, SOD, and soluble GPRX activity responded for the smaller fraction of variance that specifically defined each species at each time point. “NBT-area” was the only variable strongly correlated, i.e., correlation coefficient absolute value <0.5, with PC3. Though most of the variance (over 78%) was explained by PC1 (40.4%, a proxy for the “species” factor) and PC2 (37.8% – associated with the “time” factor), a notable 13% constituted sp. × time factor interaction-derived variance. Out of the nine parameters entered in the PCA, NBT-stained area percentage almost single-handedly represented the interaction.

## Discussion

Greater presence of vascular tissue was observed in *E. grandis* microcuttings when compared to *E. globulus* (Figures 1B–F). This may indicate delayed vascular tissue differentiation of the latter species in relation to the former. Such disparity in xylem area percentage between the two species, though not the main focus of their work, can be also observed in the data of Bryant and Trueman (2015). Also, the primitive state of vascular tissue development observed in *E. globulus*, with interfascicular cambium and stellate vascular bundle pattern (as seen in Figures 1D,E), has been previously reported (Baltierra et al., 2004). A within-species anatomical comparison of *E. grandis* × *E. urophylla* clones with varying rooting capacity, all of which were above 80%, did not reveal an association between xylem area percentage and rooting competence, although overall cutting anatomy was consistent with the patterns found in this study (Goulart et al., 2014). A general panel of *Eucalyptus* spp. rooting ability, tissue proportion, and auxin content, though elusive at this time, could help explain the variability of these traits across the genus and perhaps show phylogenetic or biogeographical links.

The increased xylem development observed in *E. grandis* when compared to *E. globulus* (Figure 1F) could be related to differences in auxin and ethylene production between these species. De Almeida et al. (2015) examined cambium-specific gene expression and auxin localization during AR development, finding that *in vitro* grown *E. globulus* had lower endogenous auxin content than *E. grandis*. Besides being a crucial regulator of AR formation, auxin is involved in xylem development. Overexpression of an impaired version of *Populus tomentosa AUXIN/INDOLE-3-ACETIC ACID* (*PtoIAA9*), an auxin-response module related to wood formation, led to lower xylem area percentages (Xu et al., 2019). Auxin has been shown to induce xylem differentiation through LONESOME HIGHWAY-LIKE 3 in *Arabidopsis* (Ohashi-Ito et al., 2013). Along with gibberellin, it has been reported as a regulator of transcriptional changes needed for xylem differentiation and secondary cell wall deposition. Auxin treatment of poplar and *Arabidopsis* led to higher xylem differentiation and reduced secondary cell wall thickness (Johnsson et al., 2019). Yet, overexpression of *YUCCA* genes, which encode enzymes on the auxin biosynthesis pathway, led to an increase in phloroglucinol-stained area, perhaps due to increased ethylene production and signaling (Hentrich et al., 2013). Several auxin analogs, when applied to *Arabidopsis*, caused an increase in xylem development rates (Yoshimoto et al., 2012). *E. grandis*, known to have higher basal auxin levels, may be more prone to xylem differentiation. The same species also had lower expression levels of an *Arabidopsis IAA12* homolog, known as a suppressor of auxin signaling (De Almeida et al., 2015). How different durations of auxin treatment could affect xylem development and lignin distribution patterns remains an outstanding question.

Phloroglucinol-stained area, largely correspondent to highly lignified xylem tissue (Figures 1C,E), was more prominently present in *E. grandis* microcuttings. However, whole-microcutting lignin content did not differ between species. Lignin content is, besides a physiologically interesting parameter, an industrially relevant trait in *Eucalyptus* trees. Although high levels of specific lignins may improve wood pyrolysis for charcoal production (de Paula Protásio et al., 2020), kraft pulping benefits significantly from low lignin content (Pilate et al., 2002). Lignin is assembled in cell walls from phenolic units by a process that naturally involves ROS generation and peroxidase activity (Tobimatsu and Schuetz, 2019).

Multivariate analysis has shown *E. grandis* and *E. globulus* to diverge in respect to redox-associated parameters in both assessed time points (Figure 5). In fact, antioxidant enzyme activity was among the main factors responsible for such different profiles (Supplementary Figure 6). ROS play vital signaling roles in plant development, often in an organ-, tissue-, cell-, and even organelle-specific manner (Mhamdi and Van Breusegem, 2018). Undoubtedly, measurement of specific reactive species can help understand global changes in the ROS pool. Still, histochemical localization of these substances, while restricted to each tissue section, may provide another dimension of understanding developmental processes. This is especially the case for a microcutting, in which a metabolic gradient is created by the excision itself (Druege et al., 2019).

Microcutting staining revealed tissue-localized patterns for superoxide and hydrogen peroxide in both species (Figures 2, 3). Superoxide anion has a very short half-life. Its unstable and radical chemical character means it will most likely attack the nearest compatible molecule within reach, i.e., Fe-S proteins, to achieve a more favorable state (Mittler, 2017). Regarding superoxide roles in AR development, Tewari et al. (2008) suggested its participation in nitric oxide (NO)-mediated adventitious rooting of Asian ginseng. Moreover, in cucumber cuttings under osmotic stress, superoxide has been shown to increase root number per explant when coupled with NO (Niu et al., 2017).

Hydrogen peroxide, in contrast to superoxide, is a non-radical ROS with a somewhat longer half-life. It is crucial for lignin polymerization reactions in cell walls. It is also directly or indirectly involved in senescence, responsiveness to stress, phytohormone homeostasis, and metabolic modulation (Petrov and Van Breusegem, 2012). It can be both a product of superoxide detoxification and a source of new, more toxic ROS, through Fenton chemistry. In fact, plant growth itself, relying on highly energetic reactions of photosystem and electron transport chain function, generates more ROS (Noctor et al., 2018; Khorobrykh et al., 2020).

Most of the redox-associated parameters assessed in this study could modulate H_2_O_2_ concentration. If DAB-stained area measurements are interpreted as the sum of all these forces, H_2_O_2_ presence in microcutting stem bases (Figure 3) is correlated with changes between Texc and Tform for both species. DAB-stained areas seemed to be associated with rapid growth tissues, such as primary xylem, phloem and epidermis. H_2_O_2_ has been shown to stimulate AR formation in mung bean (Li et al., 2009; Huang et al., 2011), ground-cover chrysanthemum (Liao et al., 2010), and marigold (Liao et al., 2011) in a dose-dependent manner. It was observed that H_2_O_2_ concentrations increase with time after excision in mung bean hypocotyl cuttings and that ascorbate, a reducing substrate for H_2_O_2_ removal, prevented AR induction (Huang et al., 2011). In poplar, optimal concentrations of H_2_O_2_ were able to accelerate AR formation, whereas higher concentrations slowed down this process and reduced root growth (Zhang et al., 2019a). ROS-mediated regulation of AR development entails auxin participation. The existence of feedback loops between auxin biosynthesis, transport, and signaling and ROS signaling has been suggested (Steffens and Rasmussen, 2016). Application of the auxin indole-3-butyric acid (IBA) promoted H_2_O_2_ production in mung bean seedlings (Li et al., 2009). Wounding induced ROS-modulated auxin biosynthesis- and transport-related genes, leading to accumulation of this phytohormone in the base of *Arabidopsis* cuttings (Huang et al., 2020).

Although broadly employed, NBT- and DAB-staining have some limitations regarding specificity, and should be viewed with caution. NBT is labile upon facing a range of molecules besides superoxide, and the exposure of its reduced form to O_2_ can regenerate NBT. DAB precipitation may occur due to higher peroxidase activity (Noctor et al., 2016). Therefore, their examination together with a pool of related parameters, such as antioxidant defenses, is advisable.

Interplay between auxin and ROS has been the focus of several studies. One of the many effects of auxin treatment is an increase in ROS generation. Moreover, auxin signaling attenuation has been shown to be affected by high levels of ROS. RAPID OXIDATIVE BURST HOMOLOG D (RBOHD) has been associated with this phenomenon, since *rbohD Arabidopsis* mutants accumulate ROS to a lesser extent (Peer and Murphy, 2013).

Upon cutting excision, a torrent of ROS is locally generated in response to tissue damage. Wound-response in cuttings is mediated by jasmonate (JA; Druege et al., 2016; Zhang et al., 2019b), and JA-mediated wound response has been associated with increased antioxidant enzyme activity (Dar et al., 2015). Such wound-induced oxidative burst has been associated with both RBOHD and peroxidase activity, and the use of enzyme inhibitors impaired AR formation in *Arabidopsis*. Also essential to ROS propagation and proper signaling were cell wall-bound peroxidases of hypocotyls (Huang et al., 2020). The present study indicated that both cell wall-bound and soluble GPRX activities strongly correlated with rooting phenotype, the former positively while the latter negatively (Figures 4D,E). This may suggest a role for the cell wall-bound fraction in signal propagation rather than as an antioxidant.

Soluble peroxidase activity increased with age in *E. globulus* microcuttings, the opposite occurring with rooting capacity and auxin content (Aumond et al., 2017). Cucumber cuttings under osmotic stress treated with the NO source sodium nitroprusside experienced higher SOD, CAT, and APX activity, which was associated with improved rooting (Niu et al., 2017). Soluble peroxidase activity induction during rooting of *Passiflora suberosa* cuttings led to lower rooting percentages (Vilasboa et al., 2020). In the current study, PCA revealed SOD activity to be strongly correlated with *E. grandis*, the easy-to-root species. Furthermore, APX and CAT activities co-variated and were associated with the formation stage of AR development. A comparison of *E. globulus* and *E. grandis* microcutting proteomic profiles revealed a substantial portion (22%) of differently abundant proteins to be redox-related. Patterns also reflected changes in proteomic profiles from the induction to the formation stage of AR development (De Almeida et al., 2020).

Nonenzymatic antioxidants have also been implicated in auxin-ROS crosstalk. Among these, flavonoids stand out for their multiple interactions with factors that play roles in this scenario. These molecules are known to interfere with auxin transport and thought to mitigate local ROS accumulation that could hinder auxin homeostasis (Peer and Murphy, 2007). The main auxin oxidation product was found at lower concentrations in *transparent testa 3 (tt3) Arabidopsis* mutants, which accumulate flavonols, whereas flavonoid-deficient *tt4* mutants had higher concentrations of the same catabolite. This indicates a possible role for flavonoids as ROS-induced auxin degradation buffers (Peer and Murphy, 2013). In a comparative study of two olive cultivars of different rooting capacity, strong positive correlation was found between total flavonoid content and rooting percentage (Denaxa et al., 2020). An increase in *E. gunnii* cutting flavonoid content has been associated with overcoming of AR recalcitrance (Di Battista et al., 2019). In the present work, the flavonoid content parameter was virtually equally represented in the “species” and “time” factors. Such diffuse response, unique in this dataset, hints at genotype- and developmental timing-related metabolic differences between the two species. These details, as is the case of many of the whole-microcutting analyses undertaken here, may be further explored by means of a detailed profiling of the stem base and its different tissues.

## Conclusion

As a first incursion into the complexity of the comparative redox status during *Eucalyptus* spp. adventitious rooting, the data from this study revealed different profiles, explained mainly by species- and time-linked variations. *Eucalyptus grandis* microcuttings showed larger xylem area than *E. globulus*. Along with this feature, SOD and both cell-wall bound and soluble GPRX activities were indicative of rooting capacity. DAB-stained area, as well as APX and CAT activities, were associated with changes from cutting excision to the AR formation stage, regardless of species. Flavonoid content was associated with both time- and species-derived variation. Overall, data indicate that *E. grandis* shows higher cambium activity and tighter modulation of redox conditions than *E. globulus*. These features may influence ROS-based signaling and phytohormone homeostasis of cuttings, thereby impacting on AR development. Besides being players in the realm of AR developmental differences, the specific features herein identified could become potential tools for early clone selection and AR modulation aiming at improved clonal propagation of this forest crop.

Future studies of AR recalcitrance will be able to further explore parameters that correlate with rooting capacity and add elements such as phytohormones and additional antioxidants, as well as other *Eucalyptus* spp. and their hybrids across the spectrum of rooting capacity. If validated under commercial greenhouse production settings and across several genotypes, some of the parameters explored in this study may contribute to the establishment of robust global models for early prediction of rooting recalcitrance, thereby contributing to germplasm selection and clonal propagation programs.

## Data Availability Statement

The original contributions presented in the study are included in the article/Supplementary Material, further inquiries can be directed to the corresponding author.

## Author Contributions

JV, CC, and AF-N contributed to conception and design of the study. JV, CC, and LR set up the *in vitro* culture system and performed biochemical analyses. JV, CC, and JM sectioned, stained, and imaged microcuttings. JV performed the statistical analysis. CC and JV wrote the first draft of the manuscript. AF-N reviewed and finalized the manuscript, and warranted financial support. All authors contributed to manuscript revision, read, and approved the submitted version.

### Conflict of Interest

The authors declare that the research was conducted in the absence of any commercial or financial relationships that could be construed as a potential conflict of interest.
