# Supplementary material for: Microcutting Redox Profile and Anatomy in Eucalyptus spp. With Distinct Adventitious Rooting Competence
Source: Front Plant Sci. 2021 Jan 20;11:620832. doi: 10.3389/fpls.2020.620832 (PMC7874081; doi:10.3389/fpls.2020.620832)
Supplement: Supplementary file 1 [file Data_Sheet_1.pdf]

Supplementary Material

Supplementary Figures

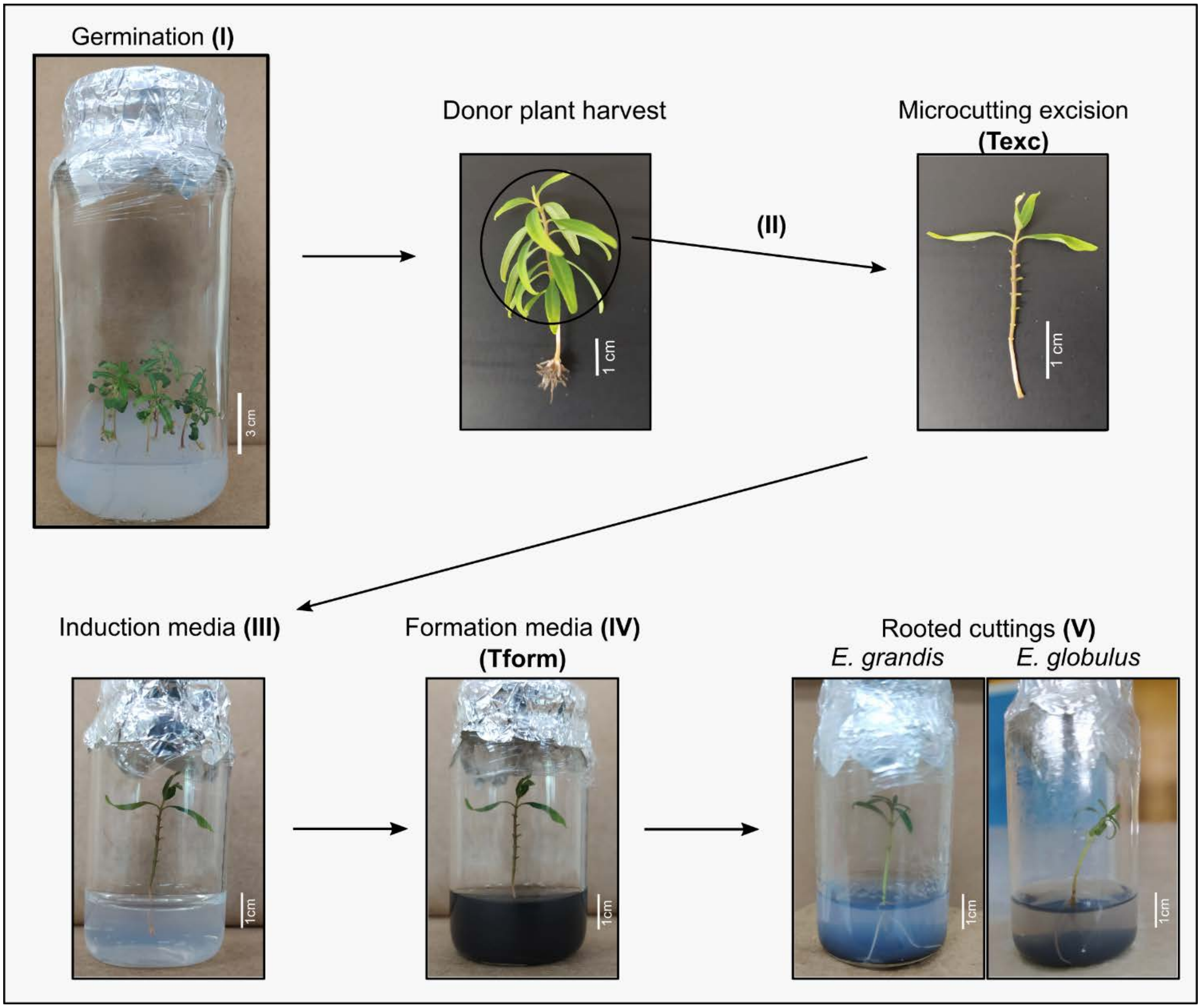

Supplementary Figure 1. Overview of *in vitro* adventitious rooting experiments with *Eucalyptus* spp. microcuttings. **(I)** *E. globulus* and *E.grandis* seeds were germinated in glass jars and seedlings were kept for 14 and 16 weeks, respectively, after which developed donor plants were harvested. **(II)** Microcuttings were obtained from the 3-cm apical portion of shoots. Other than the first two pairs, all other leaves were excised. **(III)** Microcuttings were immediately placed in glass vials with induction media for 96 h, and then **(IV)** transferred to formation media for 24 h. For biochemical, anatomical and histochemical analyses, cuttings were collected at the time of excision **(Texc)** and at five days post excision **(Tform)**. For morphological analyses, microcuttings were examined after a total of 20 days in formation media **(V)**.

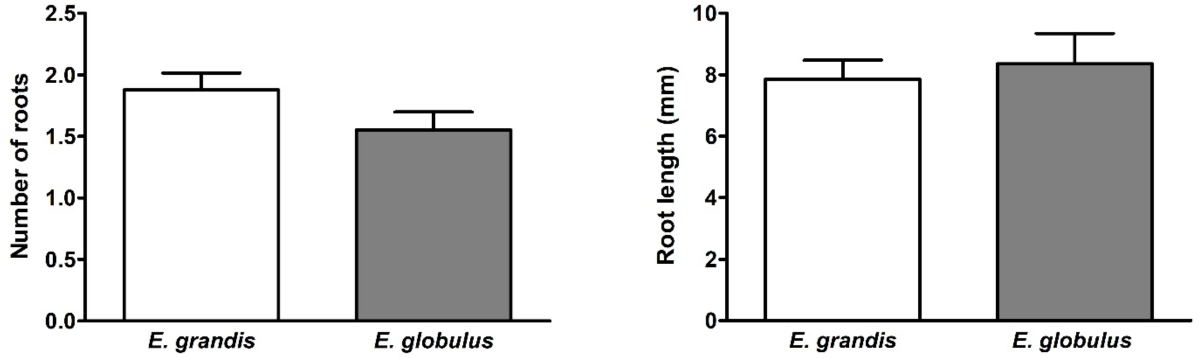

Supplementary Figure 2. Number of roots (left) and root length (right) per rooted cutting obtained from *Eucalyptus grandis* (n = 40) and *E. globulus* (n = 29) *in vitro* seedlings.

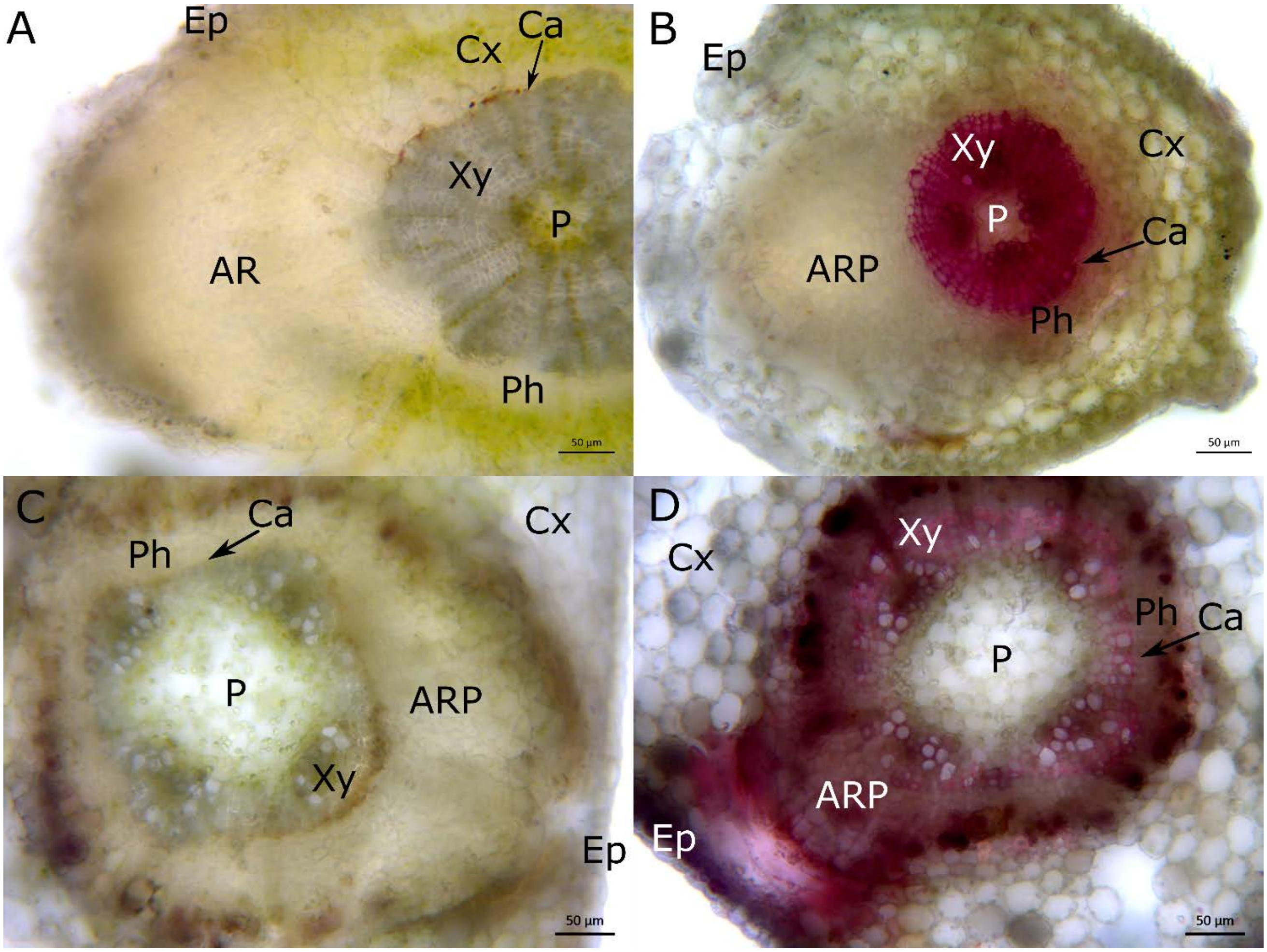

Supplementary Figure 3. Adventitious rooting in *Eucalyptus grandis* (A, B) and *E. globulus* (C, D) five days post microcutting excision (Tform). General stem base anatomy (A, C) and lignin phloroglucinol staining (B, D) (P = pith, Xy = xylem, Ca = cambium, Ph = phloem, Cx = cortex, Ep = epidermis, AR = adventitious root, ARP = AR primordium; scale bar = 50 µm).

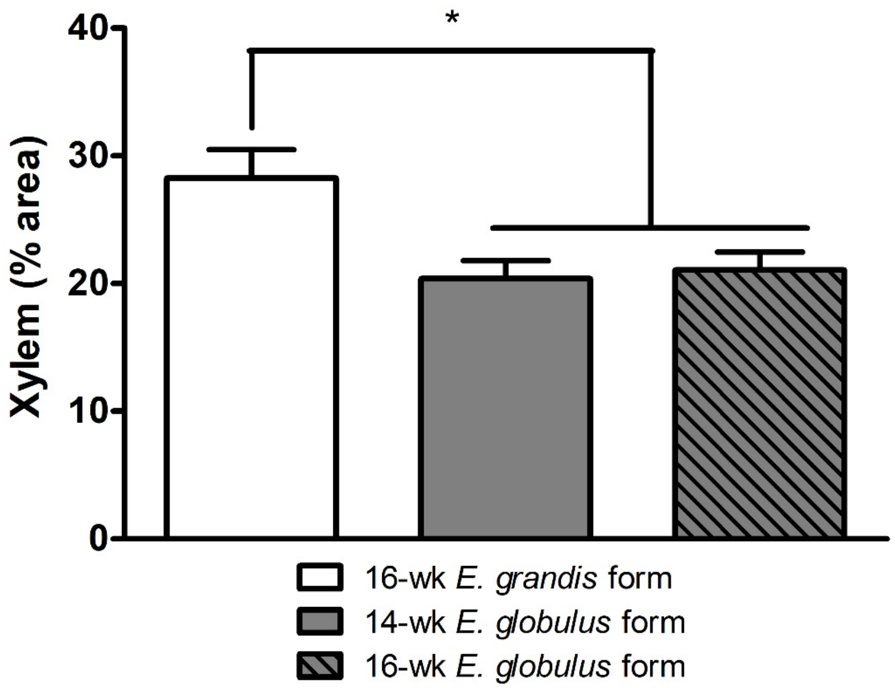

Supplementary Figure 4. Xylem area percentages measured in histological sections obtained from 16 weeks-old *Eucalyptus grandis* and 14 and 16 weeks-old *E. globulus* at the formation stage of adventitious rooting (* $p < 0.05$, paired *t*-test, n = 6)

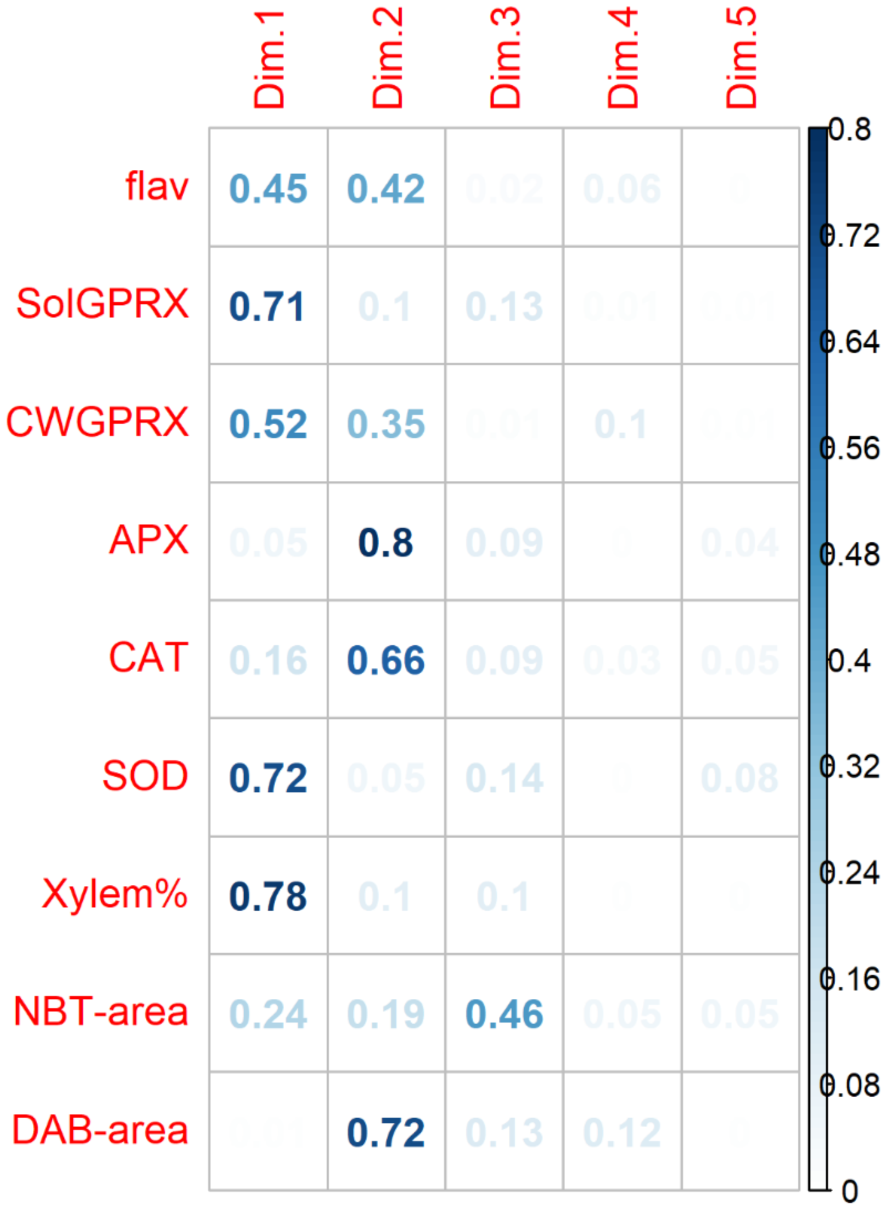

Supplementary Figure 5. Squared cosine plot of original variables against Principal Components. Values represent correlation coefficients (flav = flavonoid content, CWGPRX = cell wall-bound guaiacol peroxidase activity, Xylem% = xylem area percentage, SOD = superoxide dismutase activity, NBT-area = nitro blue tetrazolium-stained area percentage, CAT = catalase activity, APX = ascorbate peroxidase activity, SolGPRX = soluble guaiacol peroxidase activity, DAB-area = 3,3'-diaminobenzidine-stained area percentage).

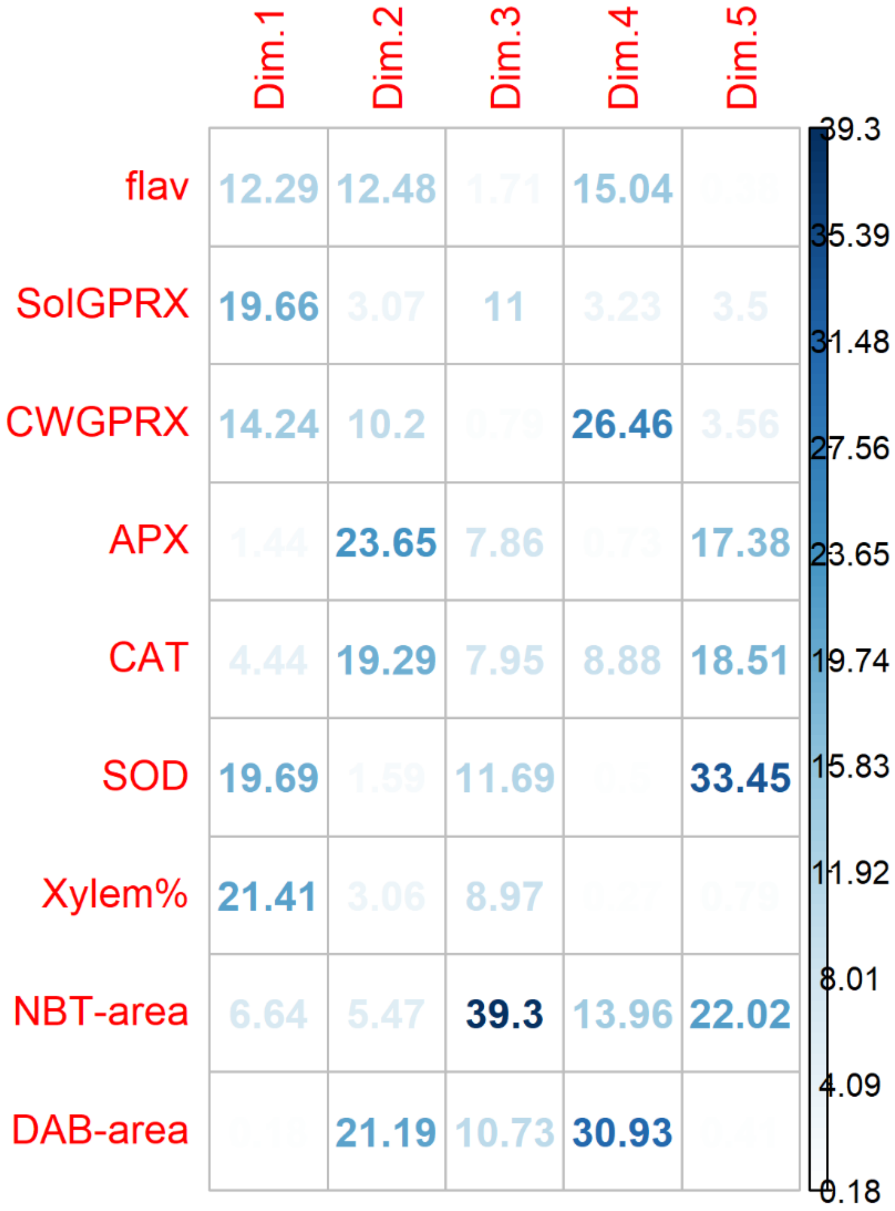

Supplementary Figure 6. Variable contribution plot of parameters against Principal Components. Values represent % contribution as a function of each $\cos^2$ value over the sum of $\cos^2$ values for each component (flav = flavonoid content, CWGPRX = cell wall-bound guaiacol peroxidase activity, Xylem% = xylem area percentage, SOD = superoxide dismutase activity, NBT-area = nitro blue tetrazolium-stained area percentage, CAT = catalase activity, APX = ascorbate peroxidase activity, SolGPRX = soluble guaiacol peroxidase activity, DAB-area = 3,3'-diaminobenzidine-stained area percentage).

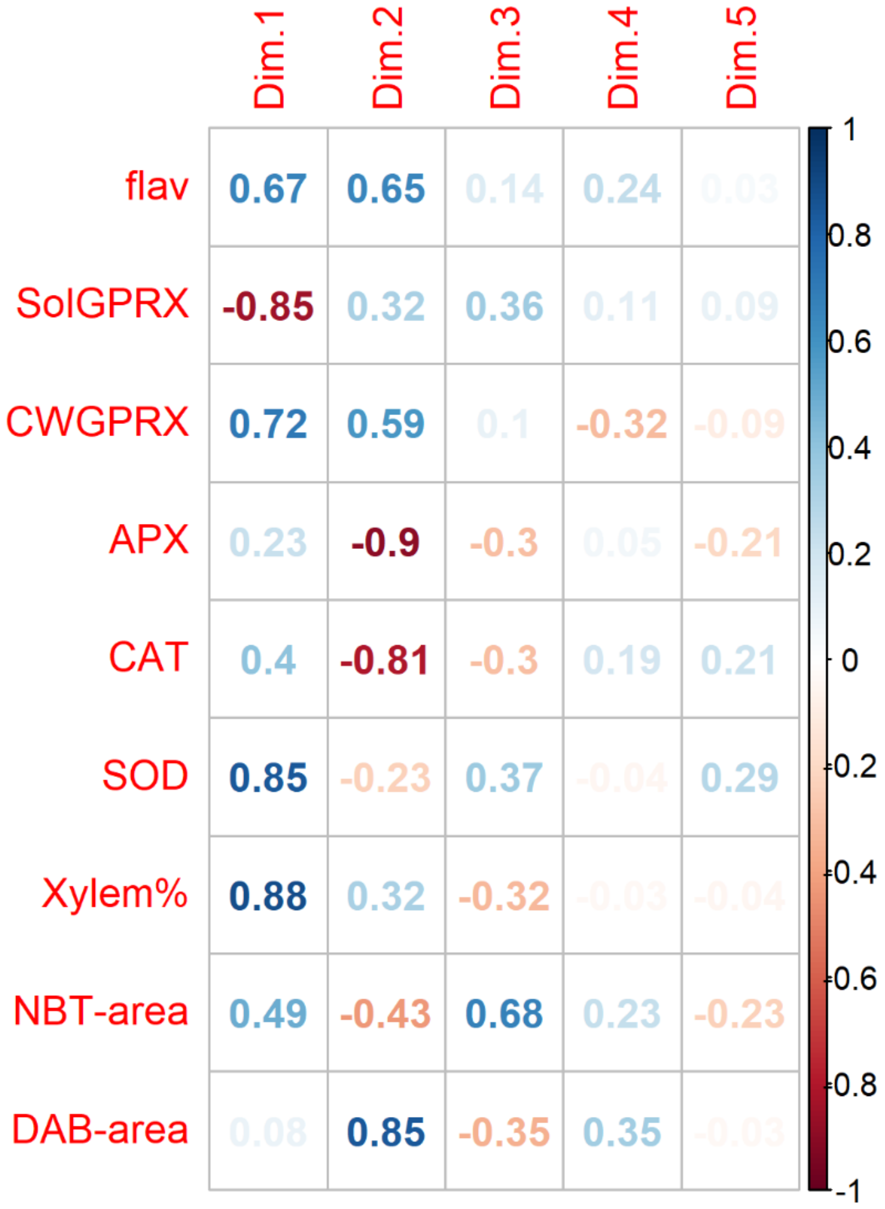

Supplementary Figure 7. Correlation plot of original variables against Principal Components. Values represent cos^2 values (flav = flavonoid content, CWGPRX = cell wall-bound guaiacol peroxidase activity, Xylem% = xylem area percentage, SOD = superoxide dismutase activity, NBT-area = nitro blue tetrazolium-stained area percentage, CAT = catalase activity, APX = ascorbate peroxidase activity, SolGPRX = soluble guaiacol peroxidase activity, DAB-area = 3,3'-diaminobenzidine-stained area percentage).
